# European Technological Sovereignty: An Emerging Framework for Policy Strategy

**DOI:** 10.1007/s10272-021-1013-6

**Published:** 2021-12-11

**Authors:** Francesco Crespi, Serenella Caravella, Mirko Menghini, Chiara Salvatori

**Affiliations:** 1 Department of Economics, Roma Tre University, Via Silvio D’Amico 77, 00145 Rome, Italy; 2 Department of Economics, Roma Tre University, Via Ostiense, 159, 00145 Rome, Italy

## Abstract

The COVID-19 crisis has revealed the deep technological and production dependencies of the EU on third countries in sectors deemed as particularly strategic and has thus fuelled the debate on (the lack of) European technological sovereignty in critical fields. This article argues that in the light of a renewed interest in relaunching a European industrial policy, technological sovereignty considerations must be fully incorporated into policy objectives and instruments.

The discussion at the EU level on the concept of technological sovereignty started before the COVID-19 crisis (Edler et al., 2020; Centro Economia Digitale, 2021). In 2019, the newly elected European Commission President Ursula von der Leyen claimed, “We must have mastery and ownership of key technologies in Europe. These include quantum computing, artificial intelligence, blockchain, and chip technologies” (European Commission, 2019a). In the same vein, the EU’s Internal Market Commissioner, Thierry Breton, declared:

> Europe cannot make its digital and green transition happen without establishing technological sovereignty. We need to work together at European level in areas of strategic importance such as defence, space, and key technologies such as 5G and quantum. In doing so, we must focus on bridging the digital gap and involving all Europe’s regions (European Commission, 2019b).

* The authors acknowledge the support from the Italian Ministry of University and Research; Scientific Research Program of National Relevance (PRIN) 2017, project “Innovation for global challenges in a connected world: the role of local resources and socioeconomic conditions”; and Centro Economia Digitale. The usual disclaimers apply.

**Francesco Crespi**, Roma Tre University; and Centro Economia Digitale, Rome, Italy.

**Serenella Caravella**, Roma Tre University; and Centro Economia Digitale, Rome, Italy.

**Chiara Salvatori**, Roma Tre University; and Centro Economia Digitale, Rome, Italy.

He highlighted how a radical change needs to be achieved quickly to manage the green and digital transitions and to avoid external dependencies in the new geopolitical context.

Outside European borders, the geopolitical context is rapidly changing, with important implications for the global economy and the distribution of technological capacities across major economic players, including the United States and China. There is a growing competition between the US and China for technological and industrial leadership not only in the configuration of global value chains, but also in geostrategic matters related to security, performance and robustness of digital networks, and international financial and payment infrastructures. In this context, major US technological companies do not have access to the Chinese market, while at the same time there are Chinese companies that are “unwelcome” in the US.

Other countries find themselves in the middle of these tensions. Europe, in particular, has structural dependencies on both the US and China in a variety of domains, from digital platforms to telecommunications infrastructure. Such dependencies call for the need to increase the level of technological sovereignty through a centralisation of efforts in strengthening technological and digital capabilities in key producing areas (Renda, 2021; Archibugi and Mariella, 2021) and an intensification of coordination activities in both decision-making and action at the EU level (Bongardt and Torres, 2021).

In this article, we argue that technological sovereignty can represent an emerging framework in which the renewed interest in European industrial policy can be incorporated (Mazzucato et al., 2015; Pianta et al., 2020). In particular, we claim that the choice of objectives (missions), strategies and instruments of industrial policy should take the actual context, constraints and ambitions (in terms of a European technological sovereignty) into account. In this respect, the paper contributes to the debate on technological sovereignty by highlighting that the public discussion cannot be limited to stating the need to achieve a greater European autonomy on the global scene, but it must define more clearly the scope of the discussion and the reasonable objectives to be achieved in order to effectively protect Europe's own interests and, above all, its values.

## Technological sovereignty: Key definitions

The term sovereignty has ancient origins. It was first developed in the 16th century by philosophers Jean Bodin and Thomas Hobbes as a way to conceptualise supreme authority over a political entity (Dunning, 1896).[1] It also assumed different meanings depending on the historical and political context; however, until the 20th century it was primarily used to express authority within a territory.[2] In this vein, sovereignty is linked to the idea that states are autonomous and independent from each other: within their own boundaries, they are free to choose their own form of government and one state does not have the right to intervene in the internal affairs of another (Krasner, 2001, 2).

More recently, as noted by Couture and Toupin (2019), the notion has been reframed in various directions that differ from earlier interpretations.[3] In particular, the concept of sovereignty has been increasingly used to describe various forms of independence, control and autonomy over digital technologies and contents; however, interpretations and definitions of this term can significantly vary.

According to its early definitions, technological sovereignty is conceived as a nationalist concept, in the sense that its goal is to promote the development of national industries and local capacity for innovation.[4]

1 It should be noted that the concept of sovereignty, albeit under different denominations, has been present since the time of Aristotle, as a fundamental principle of the national and international political order (Besson, 2012).

2 As noted by Hollis (2012), this concept of sovereignty is not limited to the landmass itself: "international law has extended the label 'territory' (and the sovereign rights and duties that accompany it) to categorize additional resources, such as the man-made infrastructure lying within a state's territory, the air space above it, mineral and oil resource below the surface, and twelve miles of the adjacent sea and seabed" (4).

3 Examples are the definition of "food sovereignty", "energy sovereignty", "body sovereignty" and "technology sovereignty" (see Couture and Toupin, 2019, 2309 and following for more details on this).

4 One of the first definitions of the technological sovereignty concept was proposed by Grant (1983), who described it as "the capability and the freedom to select, to generate or acquire and to apply, build upon and exploit commercially technology needed for industrial innovation" (239). Even earlier, as reported in Globerman (1978), the annual report of the Science Council of Canada (1977) proposes some suggestions for improving the technological capabilities of Canadian industries, pointing out that it has advocated a strategy of technological sovereignty since its first annual report in 1967. The Council defines technological sovereignty as the ability "to develop and control the technological capability to support national sovereignty" (Globerman, 1978, 42).

Though this aspect is still present, the current debate on technological sovereignty recognised that no country is able to rely only on its own capacities and market size to maintain sovereignty in a globalised and interconnected world. This suggests that sovereignty does not simply imply technological autonomy, but rather the need for a country to develop or preserve, with respect to key technologies, its own autonomy or, alternatively, to have the lowest possible level of structural dependence (Edler et al., 2020). This underlines the opportunity to avoid unilateral dependencies, especially with respect to international partners considered less reliable. Following this perspective, *technological sovereignty* can be defined as the ability of a country (or a group of countries) to generate autonomously technological and scientific knowledge or to use technological capabilities developed outside through the activation of reliable partnerships (Edler et al., 2020; ASD, 2020; Centro Economia Digitale, 2021).

Recently, the notion of technological sovereignty has been put in the context of a series of related concepts, such as strategic autonomy/sovereignty, economic sovereignty, innovation sovereignty, regulatory sovereignty and digital sovereignty. These terms are often used interchangeably (Kelly et al., 2020), a factor that contributes to confusion in the debate, avoiding a clear definition of the perimeter of objectives to be achieved and therefore the identification of appropriate policy strategies.

Even if technological sovereignty is often used indistinctly from *digital sovereignty*, the latter should be considered separately as representing the ability to act independently within the digital world.[5] This term refers to a particular form of management of the cyberspace that involves a country's control of networks and data transmitted through them. The nationality of companies that collect the largest quantities of data defines not only the strength of a country in terms of digital technological capacities, but also its full sovereignty over its digital assets.[6] Large amounts of data expose consumers' preferences and exploit them. Moreover, big data feeds and enables the application of technologies such as artificial intelligence.

There is also confusion about the interpretation of the concept of *innovation sovereignty* (Edler et al., 2020). In order to clarify the issue, it is possible to state that technological sovereignty enables the achievement of innovation sovereignty, i.e. the ability to locally exploit technologies for the development of present and future economic

5 See in this regard, with specific reference to Europe, Anghel (2020).

6 The digital business platforms on which most transactions take place are from US and Chinese companies, representing 74% and 21% of the market share respectively in the "platform business market", while Europe represents only 4% (Buest et al., 2021).

activities. In other words, scientific-technological capacities become relevant – and indeed constitute a fundamental precondition within the system – only if there are the necessary capabilities to exploit these skills from the economic-productive point of view.

If there are adequate infrastructures, institutional conditions and capacity for innovation and production, technological sovereignty contributes to *economic sovereignty*, i.e. the ability to generate value added and prosperity through independent activities or through a mutual exchange with other economies, avoiding unilateral dependencies. Economic sovereignty is based on the need for unimpeded access to natural resources and capital, as well as technologies, innovations, skills and data.

In turn, economic sovereignty also contributes to the achievement of the broader goal of *strategic autonomy*. This can be defined as the ability of a country (or a group of countries) to play an autonomous and strategic role in the geopolitical context, being an active participant in issues of global importance.[7] Strategic autonomy implies the ability to maintain independence in strategic choices while ensuring interdependencies with other countries, a necessary factor in a globalised and highly interconnected world. Once again, strategic autonomy does not imply a process of isolation or decoupling from alliances and the rest of the world, but rather describes the ability to independently pursue and manage alliances and partnerships (Bauer and Erixon, 2020).

Achieving an adequate level of technological sovereignty is a precondition for the country's strategic autonomy, as it fosters the creation of new opportunities to compete on the frontier of technological development and on international markets, with positive impacts on the country's ability to influence the global scene.

Thus, the concept of technological sovereignty proposed here does not aim at a general expansion of technological activities in areas where the country's international competitiveness is perceived to be too low. From a country's point of view, there is often an effort to achieve technological competitiveness in as many sectors as possible, however, the pursuit of technological sovereignty must take place in selected fields that are considered important according to very specific criteria.

On the other hand, it should be emphasised that the identification of crucial technologies does not provide an absolute answer as to where technological sovereignty should be achieved; it is always a choice based on considerations such as economic affordability, future risks and ease of access to imported alternatives. These considerations must be balanced against each other.

### Strategic sectors and technological sovereignty

The identification of strategic technologies is far from being straightforward. This is particularly true in a multilateral geopolitical entity, such as the EU, where the concept itself of sovereignty becomes a function of the achievement of shared objectives. According to the recently published first work programme for the European Innovation Council (2021), the strategic fields are those strictly interlinked with the EU's priorities for a sustainable, digital and healthy society. These challenges will require deep technological and innovative breakthroughs in the digital (advanced high-performance computing, edge computing, quantum technologies, cybersecurity, artificial intelligence, block-chain, cloud infrastructure technologies and technologies for the Internet of Things) and environmental (e.g. new pathways for green hydrogen production, engineered living materials) fields, as well as healthcare (artificial intelligence-driven tools for early diagnosis, point-of-care diagnostics, new approaches in cell and gene therapy, bioprocessing 4.0, health intelligence services and e-health solutions).

With regard to the pharmaceutical and healthcare sectors, the strategic content in terms of technological sovereignty can also be inferred from Germany's reaction to the recent European Commission (2020a) communication on the regulation of takeovers by foreign groups in the form of direct investments. On the basis of this communication, which urges member states to preserve their technological and industrial structures from operations that might appear hostile, the German government dissuaded the US from taking control of Curevac, a laboratory working on the development of mRNA vaccines. Meanwhile, there has been a relevant shift in the vision of the EU health policy (Greer and Jarman, 2021). In particular, to accelerate vaccine development, production and deployment by leveraging pharmaceutical research and technology, the European Commission allocated a significant budget for vaccine research through two key initiatives: the EU Vaccine Strategy COVID-19 (European Commission, 2020b) and the Pharmaceutical Strategy for Europe (European Commission, 2020c). In the latter, the European Commission (2020c) explicitly claims the need for the Union to address "unmet medical needs", highlighting, among others, the "shortcomings [that] concern the lack of development of new antimicrobials, treatments or vaccines for emerging health threats (including those

7 On the strategic autonomy concept, see among others Järvenpää et al. (2019), Leonard and Shapiro (2019), Leonard et al. (2019), Helwig (2020) and Hobbs (2020).

similar to the present pandemic, such as the severe acute respiratory syndrome coronavirus 2 (SARS-CoV-2)".

This new EU strategy follows a vaccination campaign that showed the EU to be dependent on extra-EU producers, i.e. the UK (Oxford-AstraZeneca) and the US (Pfizer, in cooperation with the German company BioNTech, Moderna and Johnson & Johnson). Moreover, this external technological dependence has caused serious delays in the development of the vaccination campaign in the EU, with major implications for the spread of the infection and its consequences in terms of hospitalisations and human lives, but also in terms of delayed economic recovery due to later reopenings compared to global competitors.

This has had a huge impact in terms of the perception among policymakers and EU citizens of the need for a European public health system (Lucchese and Pianta, 2020) as well as the relevance of European technological sovereignty (Anghel, 2020). This makes the case of COVID-19 vaccines particularly interesting.

## The use of patent indicators for the analysis of technological positioning in strategic fields

With regards to vaccine research and, more generally, to any sector considered strategic in the perspective of technological sovereignty, it can be useful to implement an analysis based on a combination of quantitative and qualitative indicators for technology intelligence. These tools are useful for assessing the distribution of technological competences as well as possible dependencies in global value chains. At the EU level, this kind of analysis allows for the determination of the extent to which member states can rely on intra-EU and/or national resources and competences and, conversely, their degree of dependence from other countries.

Relevant information in this regard is provided by patent data and derived indicators, such as patent shares and measures of technological specialisation in various technological areas. In fact, patent data allow the identification of possible technological gaps in strategic areas. This comes from the possibility to identify the specific domain in which the new knowledge has been produced through technological classes defined according to the International Patent Classification (IPC) and considering different levels of detail depending on the scope of the analysis.

For instance, with regard to COVID-19-related technologies, the World Intellectual Property Organization (WIPO, 2021) has recently provided a list of IPC classes related to technology areas that are relevant to the detection, prevention and treatment of COVID-19. This list contains several knowledge domains, covering both medical devices/equipment and medicines, and includes the IPC classes closest to vaccine research, e.g. A61K39 – medicinal preparations containing antigens or antibodies. Focusing on this specific patent class, it is possible to identify the relative technological positions of countries based on data extracted from the OECD-REGPAT database. For a better comparison between countries, information should be built on the most relevant patents, such as triadic patent families (TPFs) registered over time by the world's most important patent offices, the US Patent and Trademark Office, the European Patent Office and the Japanese Patent Office.

As it will be shown in the case of vaccine-related technologies, this kind of analysis can provide straightforward information on possible technological dependencies in key areas.

Building on patent data, a first ranking of countries in terms of their capacity to generate knowledge in this domain is shown in Figure 1, which reports the A61K39-TPF

Figure 1

**Leading countries in the generation of COVID-19 vaccine-related technologies, 2005-2016**

Country shares of A61K39 – TFPs

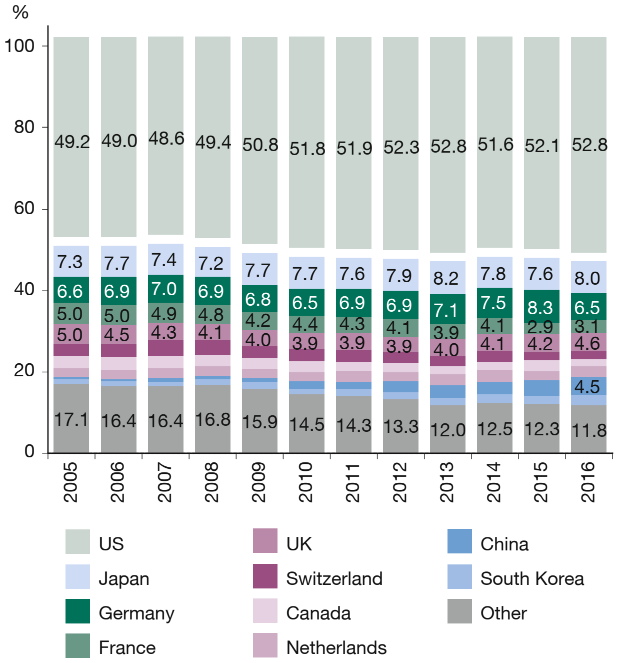

Notes: A61K39 IPC class – medicinal preparations containing antigens or antibodies. TPF – triadic patent families.

Source: Authors' elaboration based on OECD-REGPAT database, January 2021.

Table 1
**Revealed Technology Advantage index for the top 10 countries in A61K39 IPC class, 2016**

| | 2005 | 2006 | 2007 | 2008 | 2009 | 2010 | 2011 | 2012 | 2013 | 2014 | 2015 | 2016 |
|---|---|---|---|---|---|---|---|---|---|---|---|---|
| Netherlands | 24.6 | 30.9 | 38.6 | 44.2 | 39.3 | 50.1 | 47.5 | 46.7 | 49.0 | 54.8 | 49.0 | 65.6 |
| US | 32.7 | 33.3 | 33.7 | 35.2 | 38.2 | 41.2 | 42.6 | 44.0 | 47.3 | 49.9 | 53.4 | 60.3 |
| UK | 28.3 | 23.0 | 24.1 | 22.1 | 22.5 | 20.3 | 21.6 | 22.6 | 30.1 | 34.9 | 42.5 | 59.3 |
| Canada | 60.6 | 65.6 | 68.5 | 62.9 | 58.8 | 57.7 | 53.2 | 47.9 | 50.5 | 46.5 | 59.4 | 39.4 |
| France | 13.6 | 11.9 | 9.4 | 9.1 | 2.5 | 9.4 | 8.6 | 9.4 | 7.1 | 16.5 | -9.8 | 4.4 |
| Switzerland | 37.5 | 43.6 | 54.3 | 50.4 | 40.9 | 44.1 | 36.5 | 28.5 | 28.3 | 29.1 | 13.7 | -1.1 |
| Germany | -33.3 | -26.9 | -22.0 | -19.4 | -18.5 | -19.1 | -12.1 | -10.7 | -7.6 | -1.0 | 11.2 | -10.3 |
| China | -61.7 | -68.6 | -68.9 | -71.4 | -66.4 | -59.8 | -52.7 | -45.6 | -37.2 | -33.9 | -27.6 | -11.7 |
| South Korea | -84.2 | -78.0 | -77.7 | -78.4 | -78.0 | -80.0 | -79.9 | -74.8 | -73.2 | -69.5 | -68.3 | -56.0 |
| Japan | -87.9 | -87.2 | -88.4 | -89.3 | -87.8 | -88.2 | -88.7 | -87.7 | -87.6 | -89.5 | -90.4 | -90.8 |

Note: A61K39 IPC class – medicinal preparations containing antigens or antibodies.

Source: Authors' elaborations based on OECD-REGPAT database, January 2021.

country shares during the 2005-2016 period. In this case, the technological supremacy proxied by patent indicators is undoubtedly held by the US, which filled more than half of the TPFs in this class and increased its quotas from 49.2% in 2005 to 52.8% in 2016. Japan, which usually tends to slightly outperform the US in other areas, had a share between 7% and 8%, while the weight of China and South Korea appears rather marginal compared to their increasing trend of growth in other strategic fields, such as 5G and edge computing (Centro Economia Digitale, 2021). Concerning the EU, in 2016, the top performers were Germany (6.5%), France (3.1%) and the Netherlands (2.4%).

The analysis of the Revealed Technology Advantage (RTA) index (Table 1) provides complementary evidence. Positive (negative) values of RTA[8] indicate whether a country is specialised (or not) in a technology, building on the comparison between the relative frequency of patenting in a given technology, with the relative frequency of patenting in the same technology at the global level.

The US has emerged as the main player in this sector, showing a dominant position both in terms of patent shares and in terms of technological comparative advantage, with an RTA value of 60.3 in 2016. It is followed by the UK, the country of origin of AstraZeneca's vaccine, which has improved its specialisation trend since 2013, by reaching an RTA value slightly below 60 in 2016. Interestingly, Germany, which occupies the third position in terms of patent share in this class between 2005-2016, shows a negative RTA during the same period. China, South Korea and Japan record the lowest RTA values, revealing a deep de-specialisation in such a critical field. However, unlike Japan, both South Korea and, to a greater extent, China have significantly increased their RTAs in recent years.

The patent data analysis, hence, clearly provides evidence of the lack of an EU technological specialisation in the examined field, which translates into EU members' external dependence on COVID-19 vaccines.

## Towards European technological sovereignty in critical fields

US leadership in the field of COVID-19 vaccines and its implications in facing the health and economic consequences of the pandemic have paved the way for a discussion on (the lack of) European technological sovereignty in critical fields. Indeed, the strong technological and productive dependence shown by the EU with respect to the US and China in key areas (ranging from health to digital devices) translated into a particular weak condition when dealing with unpredictable and serious events, such as the current health crisis.

The analysis provided in this article suggests that in the context of a renewed interest in relaunching a European industrial policy (Mazzucato et al., 2015; Pianta et al., 2020; Archibugi and Mariella, 2021), technological sovereignty considerations should be included in the design of policy objectives and instruments. In this perspective, technological sovereignty can become a policy framework into which the priorities and targets of industrial poli-

8 The revealed technological advantage index is calculated as the share of country's patents in a particular technology field relative to the share of total patents in that country.

cies are selected and appropriate instruments designed and implemented. This requires, as a first step, a shared European understanding of what should be intended for technological sovereignty, in order to reach a shared definition of this concept at the EU level. Technological sovereignty does not imply the search for a full technological independence in all strategic fields, but the need to develop or preserve, with respect to key technologies, a certain degree of autonomy or, said otherwise, the lowest possible level of structural dependence. Thus, avoiding unilateral dependencies, especially in relation to international partners, is considered less reliable.

Once the relevant technologies from the point of view of technological sovereignty have been identified, it is necessary to carry out a qualitative/quantitative analysis to establish the positioning of EU countries in the different technological areas with respect to international competitors in order to identify strengths and weaknesses.

Hence, targeted industrial policies, supported by consistent and persistent investments in the technologies identified as critical from the technological sovereignty perspective, represent the way for achieving appropriate levels of technological sovereignty at the EU level. On the demand side, these can include the adoption of "sovereignty clauses" to be attached to innovative public procurement where necessary. Moreover, general principles could be introduced whereby public administrations must procure digital goods and services from companies that respect ethical and data sovereignty principles defined at the European level.

With regard to the digital environment, the EU should work at ensuring an advanced, secure and competitive European digital space. This would require the development of a system of coherent policies for a number of purposes related to the use of data, such as increasing the development of scientific skills, removing obstacles to the creation of a digital single market, stimulating the development of new technologies, promoting the use of new technologies (e.g. high-performance computing, artificial intelligence and Internet of Things) and competitive data and cloud infrastructures as well as establishing clear and consistent legislation, especially with regard to cybersecurity issues and data transfer.

In this area, the European Union can make a decisive contribution to the definition of rules by bringing its leadership to this field. An example is represented by the General Data Protection Regulation, where the EU has required companies around the world to comply with its privacy rules and has encouraged the development of similar regulations in other jurisdictions (even parts of the US). The EU has had a global impact on the design of the data sharing regulatory framework, demonstrating its strength in this area. Another example is provided by the eIDAS Regulation on digital identity and trust services: The construction of a common accountability and service framework in Europe has driven homogenisation in other parts of the world, such as Latin American countries, while inspiring the work of a UN working group to revise international regulation. This best practice shows how the EU can not only lead the way in the production of legislation by homogenising positions on new issues, such as the protection of personal data or electronic signatures, but can also act as a guide in regulating frontier issues, namely digital identity, blockchain, artificial intelligence and liability in human-machine interactions.

Finally, EU members have to boost their strategic autonomy in production systems by turning technological capabilities into innovative and productive capacities able to fuel international competitiveness and independence in key sectors. Actions in this direction concern supporting strategic value chains and extending their scope from industrial fields (batteries, high-performance computing and microelectronics) to services (such as digital trust) through coordinated measures on raw materials, research and innovation, investment financing, regulation, trade and skills development.
